# Disparities in Brain Cancer in the United States: A Literature Review of Gliomas

**DOI:** 10.3390/medsci5030016

**Published:** 2017-07-25

**Authors:** Dharam Persaud-Sharma, Joseph Burns, Jeran Trangle, Sabyasachi Moulik

**Affiliations:** 1 Florida International University, Herbert Wertheim College of Medicine, Miami, FL 33199, USA; jburn052@fiu.edu (J.B.); jtran009@fiu.edu (J.T.); 2 Florida International University, Herbert Wertheim College of Medicine, Department of Cellular Biology and Pharmacology, Miami, FL 33199, USA; smoulik@fiu.edu

**Keywords:** cancer, brain tumour, tumour, gliomas, neurosurgery, neurology, brain

## Abstract

In the human body, the central regulatory system of homeostasis is maintained by the brain. Its complexity is mesmerizing and many of its functions are largely uncharted. Unfortunately, its functionality is often impaired through neoplastic growths, like gliomas, which are devastating to patients and their families. Annually, gliomas are the most common primary brain tumours affecting over 20,000 people in the United States. However, despite their status as the third most common cause of cancer related death for individuals between ages 20 and 39, the aetiology of gliomas remains unknown. This paper aims to review the latest information regarding the 2016 World Health Organization (WHO) 4th edition classifications of gliomas, their malignant effects, and disparities within these classifications, as well as identify areas for further research. These suggestions for future inquiry may contribute to a better understanding of the pathology of these cancers enabling improvement in prevention, screening, and treatment.

## 1. Introduction

**Figure 1:**
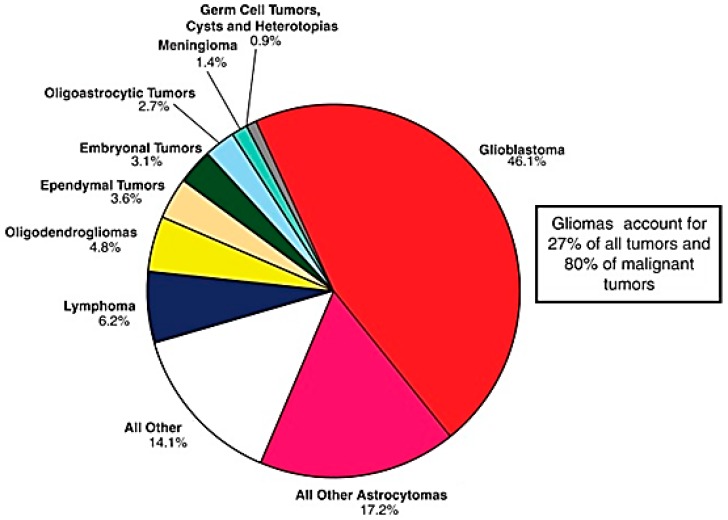
Malignant primary brain tumours by histological morphology. Adapted with permission from [1].

Malignant brain tumours represent some of the most devastating diagnoses a clinician may ever deliver to a patient. The most common malignant tumours of the brain are gliomas, accounting for over 80% of all primary brain malignancies as demonstrated in Figure 1 [1]. These cancers are aggressive, invasive, and destructive tumours, considered among the deadliest forms of human cancers [2]. “Glioma” is a general term used to characterize any tumour that originates from glial brain cells. There are three main cell types of glial origin with the potential to produce tumours. These include astrocytes leading to astrocytomas (including glioblastomas), oligodendrocytes giving rise to oligodendrogliomas, and ependymal cells forming ependymomas. Recent advances have rendered this method of classification obsolete, and the World Health Organization (WHO) 2016 4th edition classification standards include further separation of these subsections based on molecular and genetic traits.

In the United States, nearly 10,000 cases of malignant gliomas are diagnosed annually, and merely 25% of such patients survive one year after diagnosis [3]. One of the main attributes of such a poor prognosis is the lack of a standard treatment approach due to the heterogeneous nature of the tumours. Current prognosis can be affected by several factors, including the patient’s health, age, the subtype of glioma, and the location of the tumour within the brain [1,4]. Treatment is further complicated by the fact that each tumour is unique in that they can represent multiple molecular phenotypes, requiring a multi-modal approach to therapy [4]. After decades of scientific advances, aggregated data suggest that the median survival range of 9–12 months for all gliomas has remained relatively unchanged despite maximal treatment [5]. When it comes to the burden of gliomas, it is not borne evenly across the population, but varies based on race, age, and sex, among other demographic factors.

### 1.1. Gliomas, Causes, Symptoms, and Classification

**Figure 2:**
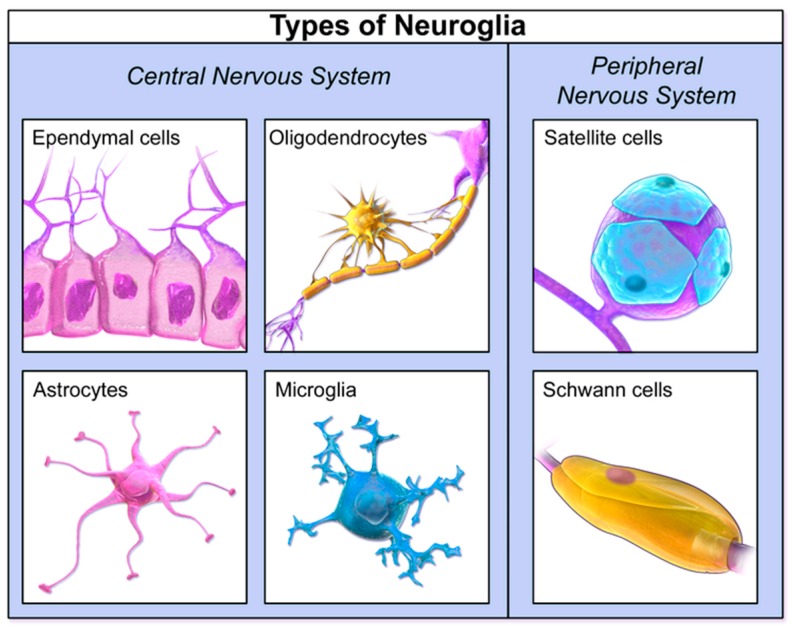
Neuroglial morphologies. Reproduced from [11].

**Figure 3:**
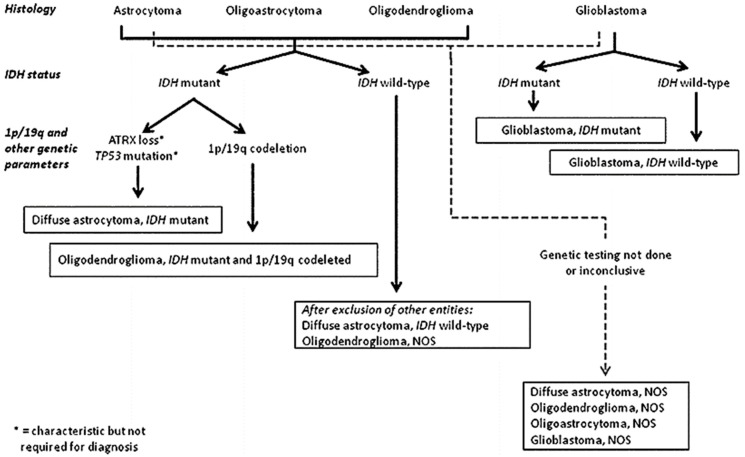
Glioma classification algorithm. Reproduced with permission from [9]. *IDH*: isocitrate dehydrogenase; 1p/19q: 1p and 19q chromosomal arms; *ATRX*: ATP-dependent helicase ATRX; *TP53*: tumour protein 53; *NOS*: nitric oxide synthase.

There are several types of gliomas in the brain, which were formerly named per the cell type they most closely resembled (Figure 2 and Figure 3). However, despite decades of validity, this classification scheme has been uprooted with the advent of molecular and genetic discoveries. Thus, beginning in 2016 with the 4th edition of the WHO Classification of Tumours of the Central Nervous System, glial cancers are now classified based on both histological appearance, as well as the aforementioned advanced genetic categorizations [6]. Among these are *IDH1/2* (isocitrate dehydrogenase 1 and 2 gene) status, 1p/19q chromosomal arm deletion status, H3-K27 mutation (a mutant histone protein), alteration of *C19MC* (a cluster of microRNA encoding genes), and *NOS* (nitric oxide synthase gene) status [6]. Regardless of the cell location, the underlying cell family from which all gliomas originate are glial cells. In the brain, glial cells function to provide nutrients, oxygen, and support to neurons. Tumours are often described as being primary-originating in the brain, or secondary-originating somewhere else in the body and metastasizing, or spreading to the brain. Malignant gliomas are the most common primary tumour of the brain.

Despite continued efforts to uncover the aetiology for these tumour types, the underlying cause remains unknown. Several models have been proposed, including a multifactorial aetiology, which also serves as an explanatory template for many other cancers. In this model, genetic susceptibilities are inherited and environmental exposures trigger mutation of critical genes thereby altering regulatory capacity of cells. This uncontrolled proliferation ultimately leads to cancer. This is similar to the Knudson two-hit hypothesis [7], although this model has not been proven with regards to gliomas. In the research of glioma pathology, several acquired genetic mutations have been identified. The most well-described genes are *IDH1*, *IDH2*, ATP-dependent helicase *(ATRX)*, tumour protein 53 *(TP53)*, *BRAF* (a proto-oncogene for protein B-Raf), and *RELA* (encodes transcription factor Nuclear Factor κB), though there are dozens more identified. Other non-single gene modifications include 1p/19q co-deletions and *RELA* or *BRAF* fusion gene products. It is important to note that such mutations are acquired and not inherited. These mutations lead to dysregulation in DNA copying, and growth causing cells to divide uncontrollably [8]. As previously discussed, with progression of discovery and detection methods, these mutations in neoplastic cells of glial origin have become critical in tumour classification, treatment, and prognosis [6].

The development of a glioma is often accompanied by a myriad of symptoms which can potentially affect any part of the central nervous system (CNS). Symptoms may include, but are not limited to headaches, seizures, visual changes, nausea, vomiting, vertigo, balance disturbances, hearing, gait, and postural instability. In other cases, patients may experience paralysis and paraesthesia of extremities, as well as respiratory arrest, coma, and death, if tumour development causes cerebral herniation.

Gliomas are classified by cell type, location, grade, and molecular markers according to the 2016 WHO guidelines [9,10]. Tumours are now classified based upon their histological and molecular or genetic composition. For example, instead of the previous distinction as astrocytoma, these tumours may now be classified as a diffuse astrocytoma, IDH1/2 mutant, diffuse astrocytoma, IDH1/2 wild-type or diffuse astrocytoma, or NOS. In addition to describing these histological features in Section 2, the function of these genetic and molecular alterations will be discussed in Section 3. Approximately 70% of gliomas in adults are supratentorial (above tentorium cerebelli), whereas 70% of the tumours in children are infratentorial (below tentorium cerebelli) [8]. Examples of the morphology of neuroglial cells are summarized in Figure 2 below.

Grading a glioma is the most important classification when determining the overall severity of malignancy. Such grading requires a biopsy of the tumour and evaluation by a neuropathologist. Generally, low-grade gliomas (WHO Grade 1 and 2) are slower growing and are associated with better prognosis, whereas high-grade gliomas (WHO Grade 3 or 4) grow faster and have a poor prognosis. While the mainstay of treatment for gliomas in most incidences is surgical resection, many gliomas typically regrow after surgical resection. Further, surgical excision of gliomas is often a challenging, complex operation that results in tremendous psychosocial and biological effects on patients and families. One notable change to the grading of brain tumours according to the WHO 2016 4th edition includes the addition of a brain invasion criterion for atypical meningiomas and the introduction of a soft tissue-type grading system. This system addresses the now combined solitary fibrous tumour or hemangiopericytoma, which is no longer graded as other CNS tumours [9].

### 1.2. Diagnosis, Treatment, and Prognosis

Patients, who present with symptoms of a glioma, are often first examined by a neurologist or a neurosurgeon. Imaging methods are used to visualize the brain tumour. Imaging techniques include X-ray, computed tomography (CT), or magnetic resonance imaging (MRI), amongst others. MRI typically provides the most detailed imaging of the brain, with the highest resolution. Confirmatory diagnosis, classification, and grading require biopsy and evaluation by a pathologist, namely, a neuropathologist.

Once diagnosed with a glioma, there are several options available to patients depending on their desired outcome. Although each glioma subtype has distinct properties and requires a tumour-specific approach, nearly all treatments include some form of surgical resection. Some patients may choose to undergo a combination therapy, while others may forego treatment. Major factors influencing these decisions are patient preferences, the anatomical location of the tumour, risks and benefits, prognosis with versus without treatment, quality of life, and grade of the glioma amongst others. Typically, supportive therapy is provided to patients for immediate alleviation of symptoms. Comprehensive therapy applies multiple therapeutic modalities, including surgery, radiotherapy, and chemotherapy. Surgery is typically highly invasive and attempts to resect as much of the tumour as possible. However, it should be noted that, in many instances, complete resection is not possible due to the orientation of vital nerves and blood vessels embedded within the tumour itself. Even if the tumour can be removed completely, there is a high tendency for regrowth [8]. Radiotherapy provides an adjuvant option, aiming to eradicate highly-proliferative cells using an ionizing beam of radiation directed at the tumour. Examples of such therapy include gamma therapy, Intensity Modulated Radiation Therapy (IMRT), Cyberknife™ (a robotic radiation therapy system from Accuray in Sunnyvale, CA, USA), and stereotactic radiosurgery (SRS), among others. Further, a combinational chemotherapy prescription is given to patients to eradicate cancer cells by exploiting the various cancer cell irregularities.

Ultimately, about half of the patients in the United States who are diagnosed with malignant gliomas of any classification are alive after one year, while roughly 25% are alive after two years [8]. The devastating outcomes of these malignant tumours indicate the tremendous need for improved treatment methods and critical research in prevention and enhanced understanding of possible etiologist.

## 2. Biology of Gliomas

### 2.1. Physiology

Glioma stem cells are self-renewing populations of stem cells with enhanced tumorigenic properties. These cells adopt many strategies to their advantage to rapidly propagate within the brain. One of these properties includes increased stem cell propagation driven by both genetic and epigenetic characteristics. Primarily, glioma cells reside in microenvironments that have limited access to nutrients, like glucose and oxygen, which are necessary for cell survival. In such low glucose conditions, glioma cells up-regulate *GLUT3* (high-affinity neuronal glucose transporter type 3) [12]. Under hypoxic conditions, glioma cells preferentially utilize HIF-2α (hypoxia-inducible factor) signalling for self-renewal, proliferation, and survival [13]. Glioma cells are also dependent on NOS2 (nitric oxide synthase-2) activity for tumour growth. These cells synthesize nitric oxide through the specific up-regulation of NOS2. Ultimately, this leads to the increased production of nitric oxide allowing for maximum oxygen acquisition by the glioma cells [14]. Glioma cells also express high amounts of VEGF (vascular endothelial growth factor) which is an important mediator of angiogenesis [15]. Clinically, immune suppression is a hallmark of cancerous growth. These cells produce a network of immunosuppressive pathways that disturb the effector function of immune cells and reduce the efficacy of therapy aimed at reinforcing immune responses against glial tumours [16].

### 2.2. Morphology

**Figure 4:**
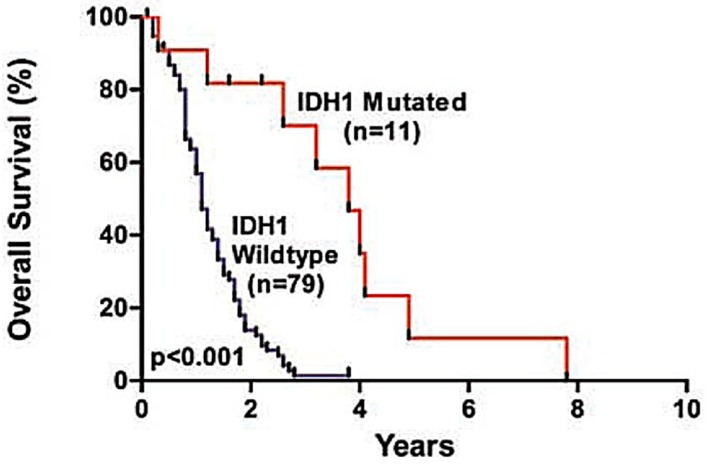
Glioblastoma survival curve by *IDH1* status. Black curve indicates survival for individuals with *IDH1* wild-type status, whereas the red curve indicates individuals with *IDH1* mutations. The *p*-Value for this relationship is less than 0.001. Reproduced with permission from [23].

**Figure 5:**
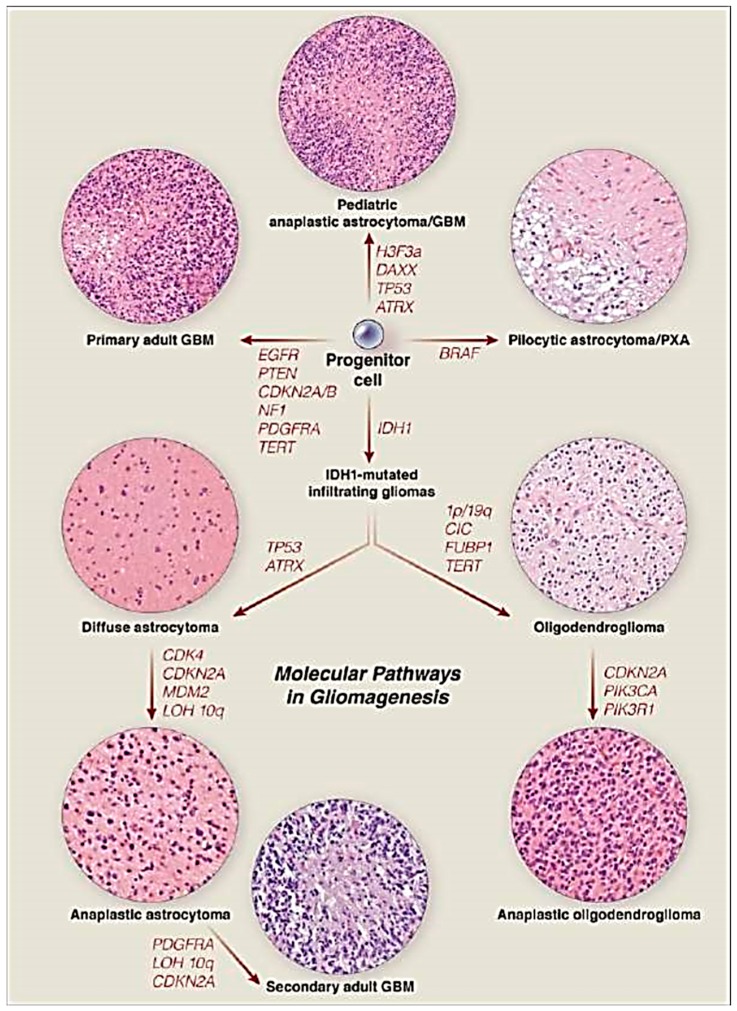
Molecular pathways in gliomagenesis. Reproduced with permission from [28]. *H3F3A:* Histone H3.3 protein; *DAXX:* Death associated protein 6; *TP53*: Tumour Protein 53; *ATRX:* ATP-dependent helicase ATRX; *BRAF*: proto-oncogene B-Raf); *EGFR:* epidermal growth factor receptor; *PTEN:* phosphatase and tensin homolog; *CDKN2A*/*B:* cyclin dependent kinase inhibitor 2A/B; *NF1:* neurofibromatin 1; *PDGFRA:* platelet derived growth factor receptor A; *TERT:* telomerase reverse transcriptase; *IDH1:* isocitrate dehydrogenase 1; 1p/19q: chr 1p, chr 19q; *CIC:* capicua transcriptional repressor; *FUBP1:* far upstream element binding protein 1; *CDK4:* cyclin-dependent kinase 4; *MDM2*:mouse double minute 2 homolog; LOH 10q: loss of heterozygosity at chr 10q; *PIK3CA:* phosphatidylinositol-4,5-bisphosphate 3-kinase, catalytic subunit alpha; *PIK3R1*: Phosphatidylinositol 3-kinase regulatory subunit alpha.

Until recently, gliomas had been classified purely on their morphological and histological features based upon the WHO first publishing of the Histological Typing of Tumours of the Central Nervous System in 1979 [17]. Iterative editions were later published in 1993, 2007 [18,19] and, most recently, in 2016 [9]. The WHO classification presented by Louis et al. primarily used microscopy and immunohistochemistry tools to differentiate glioma subtypes [19]. Biopsy specimens were categorized as astrocytomas, oligodendrogliomas, glioblastomas, or the catch-all category of oligoastrocytomas. Astrocytomas are invasive tumours that do not form a solid mass or form clear margins. Well-differentiated tumour cells exhibit a moderate increase in the number of nuclei, variable nuclear pleomorphism, and a mesh-like network of glial fibrillary acidic protein (GFAP) processes [20]. Well-differentiated astrocytomas may be identified using a GFAP immunohistochemical stain. Oligodendrogilomas (grades 2/4) are diffuse tumours comprised primarily of sheets of cells with spheroid nuclei surrounded by a clear halo of cytoplasm. Mitotic activity, nuclear anaplasia and cell density increase with increasing WHO grade (2–4) [20]. Ependymomas are often more circumscribed and less diffuse, arising from the ventricular floor. Upon biopsy, cells have regular round or ovoid nuclei with a dense fibrillary background. As seen in Figure 4 and Figure 5, ependymoma cells often form canals or perivascular pseudorosettes, with tumour cells arranged surrounding central blood vessels [20]. Less well differentiated tumours were historically difficult to accurately identify, and determining astrocytic or oligodendrocytic cell lineage was a major challenge, leading to the establishment of the oligoastrocytoma classification. Studies have shown that poorly-differentiated tumour cells have high rates of inter-observer variability leading to misdiagnosis and inappropriate clinical treatment [21]. With the advent of molecular genetic techniques and identification of key prognostic markers, tumour classification is rapidly improving [9].

### 2.3. Genetics

The recent WHO classification is the first to incorporate molecular parameters to further define gliomas in an objective manner [9]. Several molecular genetics studies have been conducted identifying consistent gene loci anomalies associated with particular types of cancer. Not only are these genetic markers used to classify glioma subtypes, but they also are often prognostic predictors of the course of the disease. The most common mutations identified to date are in the *IDH1/IDH2* genes with *IDH1* mutations identified in approximately 80% of grade 2/3 gliomas [22]. The most common mutation is a single amino acid missense mutation in *IDH1* at arginine 132 (R132H), identified in 12% of samples [23]. Glioma survival is strongly associated with the *IDH1/IDH2* mutation, with *IDH1* wild-type typically being associated with poorer outcomes [24,25]. Another molecular marker identified is co-deletion of the 1p and 19q arms of their respective chromosomes [24]. Use of *IDH1* mutation and 1p/19q co-deletion status has nearly eliminated the previous amorphous classification of oligoastrocytoma [26]. With the cost of whole-genome sequencing falling, numerous other genetic associations have been identified, including *TP53, ATRX, BRAF,* and *RELA* [25]. We are nearing an age where every tumour is sequenced, and a comprehensive library of genomic modifications will inform behaviour, clinical course, and therapeutic targets.

### 2.4. Genes

Since the completion of the human genome project, the past fifteen years have yielded several target genes associated with the development and progression of gliomas. *IDH1* and *IDH2* genes mutations play a prominent role in gliomagenesis, specific to both oligodendrogliomas and astrocytomas. *IDH1/2* are found on chromosome 2 and mutations are found in upward of 80% of low grade gliomas, with 93% of those mutations occurring in *IDH1* [22,27]. Rather paradoxically, as shown in Figure 4, the *IDH1* wild-type gene actually confers worse prognosis in glial cell tumours, being associated with WHO grade 3/4 glioblastoma, whereas, *IDH1* mutation confers a better long-term prognosis. A monoclonal antibody that binds to the R132H mutated *IDH1* gene allows for easy testing of biopsy specimens. *IDH1* mutations are also associated with acute myeloid leukaemia (AML) [22,27].

Whole arm deletions of the 1p and 19q chromosomes are diagnostic for oligodendrogliomas, and confer a better prognosis [26]. Standard testing consists of fluorescent in situ hybridization (FISH) to identify loss of chromosomal genetic material. The *ATRX* and *TP53* genes are both strongly associated with astrocytomas, often co-occur with *IDH1/2* mutations, and do not occur with 1p/19q deletions. *ATRX* is a chromatin regulatory gene and *TP53* is a tumour suppressor gene. Mutations to the *BRAF* gene, primarily through fusion to *KIAA1549* gene, are present in a majority of pilocytic astrocytomas (Figure 5). Point mutations in the *BRAF* gene are strongly associated with pleomorphic xanthoastrocytomas, primarily at the V600E site, in which valine is replaced by glutamic acid at amino acid 600. *RELA* fusion products with *C11orf95* are found in over 70% of ependymomas.

## 3. Glioma Classification

### 3.1. Diffuse Astrocytoma and Oligodendroglioma

The WHO 2016 4th edition includes a new category of “diffuse astrocytic and oligodendroglial” tumours, combining tumours with similar phenotypic (“diffuse” or invasive) and genetic characteristics under a single category. Formerly, tumour cell histological markers separated tumours based on presumed lineage—with astrocytic and oligodendroglial tumours being wholly separate. This effectively increases the category’s prognostic meaning as it more closely aligns to tumour behaviour and tracks more closely to outcomes. This tumour category includes a broad array of subcategories including diffuse astrocytomas, anaplastic astrocytomas, *IDH* wild-type and *IDH* mutant glioblastomas, diffuse midline gliomas, oligodendrogliomas, and anaplastic oligodendrogliomas—with many phenotypic categories being even further delineated with genetic markers [6].

#### 3.1.1. *IDH*-Mutant Tumours

The *IDH* mutation is one of the major new genetic criteria differentiating tumours into different types. While there is no single “*IDH*-mutation” or “*IDH*-wild-type” category—*IDH* genetic status when coupled with other phenotypic information can not only narrow the tumour into its classification category, but also inform the course of the disease and optimal treatment. *IDH* mutant tumours include diffuse astrocytic, anaplastic astrocytoma, glioblastomas, 1p/19q co-deletion oligodendrogliomas, and anaplastic oligodendrogliomas [6].

**Figure 6:**
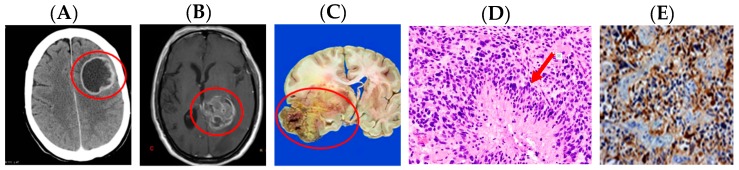
Comparative imaging and histological features for glioblastomas (Formerly Multiforme—3rd edition World Health Organization (WHO) Classification). Arrows and circles added to assist with identification of pathology. (**A**) Glioblastoma—axial computed tomography (CT) image. Modified from [31]; (**B**) glioblastoma—axial cross section T1 weighted magnetic resonance imaging (MRI) image. Modified from [31]; (**C**) glioblastoma (formerly multiforme)—gross pathology specimen. Modified from [31]; (**D**) pseudopalisading with arrow pointing to central necrosis of glioblastoma cells—haematoxylin and eosin (H&E) stain [32]; and (**E**) astrocytic lineage cells with glial fibrillary acidic protein (GFAP) marker on immunohistochemistry stain brown. Reproduced from [33].

Astrocytomas are tumours arising from astrocytes. *IDH*-mutant astrocytomas include anaplastic and diffuse phenotypes. These cells are the most numerous glial cells in the CNS. This diverse cell type varies in both morphology and function as shown in Figure 6. Typically, astrocytes serve as stem cells; they define the brain microarchitecture, control potassium homeostasis, control synaptic maintenance, and control local blood flow to provide neurons with nutritional support [29]. Diffuse astrocytomas are malignancies that invade the surrounding tissue, but grow slowly. They usually contain cysts and mucus. These tumours arise in many areas of the CNS including the cerebellum, cerebrum, brain stem, and spinal cord. Most diffuse astrocytomas contain an *IDH*-mutation, and since only a few common *IDH* polymorphisms are regularly tested for, it is conceivable that even some *IDH* wild-type tumours are, in fact, *IDH* mutants, just at a previously unidentified locus [9]. *IDH* mutations also arise in oligodendrogliomas, which are discussed in Section 3.1.4.

Patients may present with headaches, seizures, memory loss or behavioural symptoms. These cancers more frequently affect males over age 45. For all subtypes, surgery is the first procedure undertaken to remove the tumour, though regrowth after excision is a common observation. Subsequent radiation may be used to target areas of incomplete removal [30].

#### 3.1.2. Nitric Oxidase Synthase Mutation

NOS is a molecule that enhances vascular relaxation effects, macrophage and neutrophil phagocytosis and cyclic guanosine monophosphate (GMP) production [34]. This factor is relevant in the development of gliomas as it is a potent mediator of angiogenesis by stimulating VEGF production. Studies have demonstrated that NOS is constitutively active in certain mutated astrocytomas. It has been demonstrated that, at higher tumour grades, NOS is found at higher concentrations [35]. This is to say; *NOS* mutation increases the likelihood that patients may develop gliomas through potentiation of neovascularization and also attributes poorer prognosis for those afflicted with *NOS* mutated tumours. Further, the dilatory effect of NOS allows for maximal oxygen uptake and utilization by glioma cells [14]. The presence of *NOS* mutations is now used to further differentiate diffuse gliomas, anaplastic astrocytomas, glioblastomas, oligodendrogliomas, oligoastrocytomas, and anaplastic oligoastrocytomas [6]. As molecular technologies and targeted drug delivery models are developed, this molecular alteration may become one possible means of treating and eliminating tumours of glial origin.

#### 3.1.3. H3-K27M Mutation and Diffuse Midline Gliomas

Diffuse midline gliomas (DMGs), are highly malignant midline gliomas that are primarily of astrocytic differentiation. In a 2012 study, it was shown that two somatic mutations of the *H3F3A* and *HIST1H3B* genes of histone H3, encoding the H3.1 and H3.3 histone variants are seen in paediatric diffuse intrinsic pontine gliomas (DIPG) and non-brain stem gliomas [36,37,38]. Of particular note, the K27M mutation (K-lysine to M-methionine at amino acid 27) was present in many H3 variants of both DIPG and thalamic glioblastomas. Further research reveals that the H3-K27M mutation is present in a majority of high-grade infiltrative astrocytomas arising within midline structures (thalamus, pons, spinal cord) in both paediatric and adult cases [38]. Comparatively, a G34R/V (G-glycine to R-arginine/V-valine at amino acid 34) was detected in a minor subset of peripheral glioblastomas in the cerebral hemispheres. Clinical studies have shown that DIPGs with H3-K27 mutations are associated with an aggressive clinical behaviour and poor prognosis regardless of histological grade, thus, assigned a grade IV designation in the WHO 2016 4th Edition Classification [38,39,40,41,42].

#### 3.1.4. 1p/19q Co-Deletion

**Figure 7:**
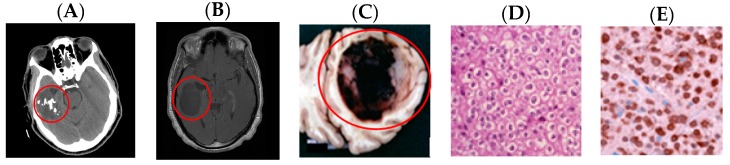
Comparative images of oligodendrogliomas. Arrows and circles added to assist with identification of pathology. (**A**) Left frontal oligodendroglioma—axial CT. Modified after [48]; (**B**) left frontal oligodendroglioma—axial fluid attenuated inversion recovery (FLAIR) MRI. Modified after [49]; (**C**) oligodendroglioma—gross pathology specimen. Modified after [50]; (**D**) round nuclei with surrounding clear cytoplasm characteristic of oligodendrogliomas—H and E stain. Reproduced from [51]; and (**E**) R132H+ immunohistological stain specific to *IDH1* mutation. Reproduced from [50].

Oligodendrogliomas are tumours arising from oligodendrocytes. The typical function of oligodendrocytes is to form myelin in the central nervous system [43]. The 1p/19q co-deletion is one of the best documented genetic markers for gliomas and is required to be deemed an oligodendroglioma (anaplastic or typical) [9]. Oligodendrogliomas tend to be a glioma category with some of the best outcomes and highest survival rates [24]. As shown in Figure 7, these tumours are soft and often contain calcifications with small areas of haemorrhage or cyst development. This type of tumour is most common in adult males between ages 35 and 44. They are rarely seen in paediatric populations, accounting for only three percent of primary brain tumours [44]. These tumours may cause seizures, headaches, personality changes, unilateral muscle weakness, or difficulty with short-term memory. Factors that may increase the risk of oligodendroglioma include a family history of brain tumours, history of epilepsy, and head trauma [45]. Surgical excision is the primary intervention in the treatment of oligodendrogliomas. Subsequent chemotherapy may be adjuvant to surgery in anaplastic tumours, mixed tumours, or those with 1p or 19q deletions. Since *IDH* mutant tumours tend to progress more slowly, management is often less aggressive [46]. Similarly, depending on molecular markers and grade, radiation may be indicated for treatment of oligodendroglioma. Favourable prognostic indicators include frontal lobe location, low WHO grade, and combined 1p/19q loss [47].

### 3.2. Other Astrocytic Tumours

Other tumours in the oligoastrocytoma family include pilocytic astrocytomas and subependymal tumours. Pilocytic astrocytomas almost never metastasize. They may form cysts or may be included within a cyst [52]. Pilocytic astrocytomas affect children and young adults [52]. Subependymal giant cell astrocytomas are ventricular tumours associated with tuberous sclerosis [6]. Anaplastic xanthoastrocytomas tend to have tentacle-like projections that invade neighbouring tissues, making surgical excision difficult [6]. Chemotherapy may be used in anaplastic xanthoastrocytoma, especially in children, to avoid radiation exposure [30]. In pilocytic astrocytomas, partial resection, optochiasmatic location, invasion, or surrounding structures and pilomyxoid morphology are associated with a worse prognosis [52].

### 3.3. Ependymal Tumours

Ependymomas arise from the ependymal cells that comprise the epithelial lining of the ventricles of the brain. The tumours are generally soft and may contain calcifications or cysts. They were formerly classified by location and now include division based upon *RELA* fusion status [6]. These tumours generally develop supratentorially arising above the tentorium cerebelli, a thick membrane separating the top two-thirds of the brain from the bottom third.

**Figure 8:**
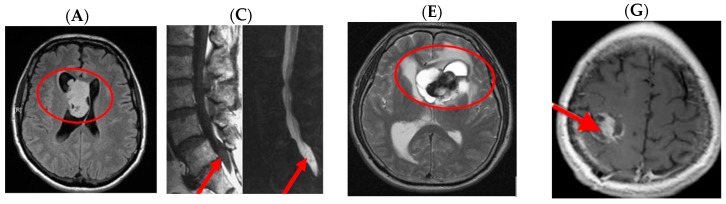

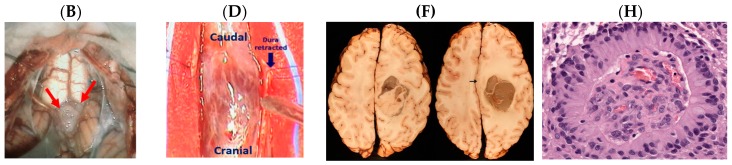
Ependymoma subtypes. Arrows and circles added to assist with identification of pathology. (**A**) Subependymoma pushing into the lateral ventricle—T2 FLAIR axial cross section MRI image. Modified after [53]; (**B**) gross specimen, arrows pointing to subependymoma pushing into fourth ventricle. Modified after [54]; (**C**) myxopapillary ependymoma in caudal spinal cord—CT. Modified after [55]; (**D**) myxopapillary ependymoma near cauda equine—gross specimen. Reproduced from [56]; (**E**) ependymoma in left lateral ventricle—T1 weighted axial cross section MRI image. Modified after [57]; (**F**) large ependymoma encompasses entire fourth ventricle—gross specimen. Reproduced from [58]; (**G**) anaplastic ependymoma—CT image. Modified after [59]; and (**H**) ependymoma pseudorosette encroachment on neighboring blood vessels. Reprodcued from [60].

This supratentorial region contains the cerebral hemispheres, as well as the lateral and third ventricles. Infratentorial ependymomas occur below this membrane, arising from the fourth ventricle in proximity to the brainstem and cerebellum. As shown in Figure 8, several of these features are observable on MRI. In Figure 8A, an infratentorial ependymoma in the posterior fossa is shown on a sagittal MRI image. Here, the tumour has been circumscribed in red for identification. An axial view of an ependymoma is also shown in Figure 8B. This image illustrates an ependymoma of the left lateral ventricle. Expansion of an infratentorial mass is able to compress the thalamic regions of the brain, causing multiple neurosensory and motor deficits.

**Figure 9:**
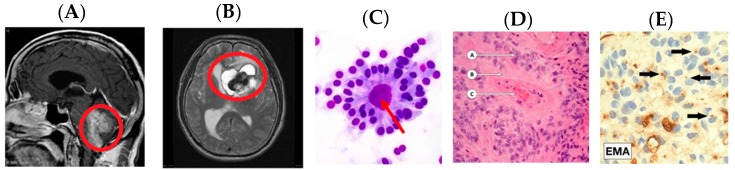
Comparative imaging and histological features for ependymomas. Modified after [57]. Arrows and circles added to assist with identification of pathology. (**A**) Posterior fossa, infratentorial image of an ependymoma—Sagittal T1W MRI; (**B**) axial view of a lateral ventricle ependymoma glioma—Axial T2 weighted MRI; (**C**) ependymoma “True” rosettes on histology with tumour cells aligning around an empty core (lumen); pathognomonic for ependymomas—H and E Stain; (**D**) ependymal pseudorosettes: A–Ependymal cells tumour cells, B–Perivascular clearing C–Blood vessel, and (**E**) epithelial membrane antigen (EMA) stain—black arrows pointing to EMA positive brown cytoplasmic vacuoles representing “microlumens”.

Comparative histological images of these tumours are shown in Figure 9D,E. Figure 9C, shows an ependymomal “true” rosette, comprised of primitive tumour cells surrounding an empty lumen, assembling as if around a ventricle. Though rarely seen, they are pathognomonic of ependymomas. Other ependymoma histologic features include perivascular pseudorosettes, with ependymal tumour cells surrounding a central blood vessel, and ependymal canals with columnar tumour cells forming a column of cells around a central lumen.

In adults, the location of ependymomas differs from that of the paediatric presentation. In children 90% are found in the brain whereas, in adults, 60% are found in the spinal cord [61]. Patients may present with back pain, sensory deficits, weakness, bowel or bladder dysfunction, mental status change, lack of coordination, vision changes, or seizures. Little is known about the cause of ependymoma; however, there is an association with neurofibromatosis type II [62]. After visualization with CT or MRI, surgery and radiation therapy are indicated for patients with ependymomas. Tumours that can be grossly removed have the best postoperative diagnosis. In patients presenting with hydrocephalus, implantation of a ventriculoperitoneal shunt is indicated. Radiation is usually used in adolescent or adult patients. Chemotherapy may be used in children to delay radiation treatment due to the long-term side effects [61]. Generally, outcomes are worse for younger children with advanced disease that cannot be resected. For this reason, advancement in tumour control in these patients remains a critical research frontier [63].

### 3.4. RELA

Recent amendments to the classification scheme for ependymomas rely on the presence of *RELA* fusion positivity [6]. This mutation confers constitutive activation of NFκB (nuclear factor-κB) family of transcriptional regulators. In this manner, these cells constantly act as mediators of the cellular inflammatory response [64]. Though it is poorly understood how this mechanism precisely confers a survival advantage to tumour cells, the presence of this mutation is confirmed in nearly two-thirds of ependymomas [64]. This genetic alteration is detectable using FISH, making it readily measurable in clinical practice. Further, the presence of *RELA* mutations is correlated with the expression of L-1 cell adhesion molecules (CAM). *RELA* is not necessarily ependymoma-specific, but may guide treatment in the future as novel molecular and gene targeted therapies are developed [64].

### 3.5. Other Gliomas

A few very rare glial cell tumours lack any differentiating mutations and are described primarily on histologic and phenotypic features. These tumour types include angiocentric gliomas, astroblastomas, and chordoid gliomas of the third ventricle [9]. Angiocentric gliomas are quite rare, with less than 100 described in the literature through 2016 [65]. They are described as slow-growing, supratentorial tumours that are often accompanied by seizures [66]. Treatment primarily consists of resection and is curative, although given the low incidence of this tumour type approaches often vary. Astroblastomas are another rare tumour that occurs primarily in children with seizures. More commonly occurring in females, they tend to recur frequently with surgical resection being the most common treatment approach [67]. Chordoid gliomas of the third ventricle typically occur around age 50 and have an indolent course. Their location, however, frequently lies in close proximity to the hypothalamus making even their slow-growth dangerous, often requiring surgical resection—a delicate procedure [68]. 

### 3.6. Neuronal and Mixed Neuronal Glial Tumours

Another major alteration to the WHO guidelines for the classification of brain tumours, include two lesions: diffuse leptomeningial glioneuronal tumours (DLGNT) and multinodular and vacuolating neuronal tumours (MVNT). According to Komori [6], DLGNT tumours include characteristic involvement of the leptomeninges of the spinal cord with or without parenchymal invasion [6]. These tumours include a dominant oligodendroglia-like cell mass and often possess *BRAF* fusions and chromosome 1p deletions [6]. Comparatively, MVNT tumours possess multiple nodules composed of vacuolating dysplastic neurons in the subcortical white matter. Hamartomatous MVNTs include a distinctive nodular or ribbon-like growth pattern [6]. 

## 4. Trends in Gliomas in the United States

### 4.1. Racial Trends in the United States

**Figure 10:**
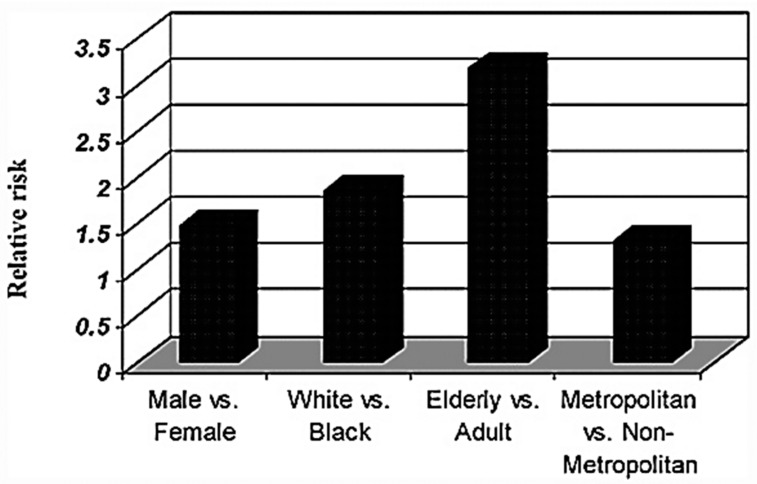
Relative risk of glioma diagnosis per the demographics in the United States (1973–2001, Surveillance, Epidemiology, and End Results SEER Registries). Reproduced with permission from [69].

The incidence of brain cancer has steadily increased for the last three decades, but has begun gradually trending downwards in recent years. However, the diagnosis of glioblastoma is rising. Given its poor survivorship, this trend is particularly concerning. Evaluation of data from 1973 to 2001 suggests that the most vulnerable demographic for the diagnosis of any brain cancer are white, elderly white men residing in a metropolitan area, as depicted in Figure 10, although comprehensive studies are limited [69]. Regarding glial cancers, data from 1992 through 2007 suggests significant findings regarding race and the diagnosis of glioblastoma versus other glial brain cancer. In the United States, Blacks, Asian/Pacific Islanders, and American Indians exhibit substantially lower rates of both diagnoses. However, the variation in this rate was less dramatic in non-glioblastoma glial cancers than glioblastoma [69].

### 4.2. Age and Sex

As illustrated in Figure 10, brain cancer diagnoses are more frequently observed in elderly populations [69]. Glioblastoma diagnosis increases in proportion to age. Glioblastoma increases at an exponential rate with age, whereas non-glioblastoma increases in a pattern that is approximately the square root of age. This suggests that glioblastoma is likely developed by large amounts of genetic or epigenetic changes that accumulate over time, whereas non-glioblastoma may arise due to a small number of changes or slow expansions of premalignant changes. This hypothesis is supported, as glioblastomas demonstrate more complex molecular changes than non-glioblastoma cancers. The rate of diagnostic frequency changed at a similar rate for both males and females. However, Asians/Pacific Islanders and American Indians had the slowest growth of this rate [69].

Brain cancers are more commonly diagnosed in men [69]. The incidence of glioblastoma and other glial cancers is higher for males than females. However, the rate is more similar in non-glioblastoma than glioblastoma (1.4 vs. 1.6). In Hispanic Whites, however, this association is not observed. This rate is most pronounced in the peri- and postmenopausal range, suggesting that reproductive hormones may be involved in the development of these cancers [69].

### 4.3. Social Determinants

It has been suggested that widespread use of CT beginning in the 1970s may contribute to the increasing date of diagnosis of brain cancers and glioblastoma [69]. This may be associated with the most frequently diagnosed demographic previously described, as the individuals most likely to have access to routine CT scans are white males in metropolitan areas. This is to say that those of lower socioeconomic status, or that reside in rural areas, may present with artificially reduced rates of diagnosis due to inadequate diagnostic imaging availability or referral for such services. This may be further complicated by the lack of access to appropriate providers in non-metropolitan areas. Inability to obtain CT imaging due to lack of transportation, cost, or insufficient insurance coverage may skew the data in the manner described.

## 5. Conclusions and Future Work

In this paper, an overall evaluation and summary of currently available knowledge regarding gliomas has been made to include the most common clinical presentations, known molecular pathology, diagnostic imaging, social demographics, physiology, as well as gross pathology and histology. Despite having a general understanding of evident pathological gliomas, this knowledge represents only a fraction of information yet to be determined. The continued poor prognosis for patients despite the best available treatments and detection methods elucidates the need for a substantial amount of work needed to enhance earlier detection and therapies to improve long-term prognosis. The current trends in research are now focused on addressing these needs, and one of the most promising research sectors dually serving to accomplish these objectives lies within molecular pathology. In addition, brain tumour development is a very complex process and cannot be overly simplified. However, current physiological models do not provide a clear algorithmic mechanism by which these cancers develop. Part of this complexity lies within the multifactorial nature of these proliferations and the undetermined cellular pathophysiology. By coupling further epidemiological investigation with continued basic science exploration, it is promising that a discovery of the possible aetiologies can be made with a high degree of sensitivity and specificity. This may drastically improve the timeliness of diagnosis and the long-term prognosis for those who develop tumours.
